# Eating and hypothalamus changes in behavioral-variant frontotemporal dementia

**DOI:** 10.1002/ana.22244

**Published:** 2011-02

**Authors:** Olivier Piguet, Åsa Petersén, Bonnie Yin Ka Lam, Sanaz Gabery, Karen Murphy, John R Hodges, Glenda M Halliday

**Affiliations:** 1 Neuroscience Research AustraliaSydney, Australia; 2 School of Medical Sciences, University of New South WalesSydney, Australia; 3 Translational Neuroendocrine Research Unit, Department of Experimental Medical Science, Lund UniversityLund, Sweden

## Abstract

**Objective:**

Behavioral-variant frontotemporal dementia (bvFTD) is a progressive neurodegenerative brain disorder, clinically characterized by changes in cognition, personality, and behavior. Marked disturbances in eating behavior, such as overeating and preference for sweet foods, are also commonly reported. The hypothalamus plays a critical role in feeding regulation, yet the relation between pathology in this region and eating behavior in FTD is unknown. This study aimed to address this issue using 2 complementary approaches.

**Methods:**

First, 18 early stage bvFTD patients and 16 healthy controls underwent high-resolution structural magnetic resonance imaging and assessment of eating behavior. Hypothalamic volumes were traced manually on coronal images. Second, postmortem analyses of 12 bvFTD cases and 6 matched controls were performed. Fixed hypothalamic tissue sections were stained for a cell marker and for peptides regulating feeding behaviors using immunohistochemistry. Stereological estimates of the hypothalamic volume and the number of neurons and glia were performed.

**Results:**

Significant atrophy of the hypothalamus in bvFTD was present in both analyses. Patients with high feeding disturbance exhibited significant atrophy of the posterior hypothalamus. Neuronal loss, which was observed only in bvFTD cases with Tar DNA protein-43 deposition, was also predominant posteriorly. In contrast, orexin (hypocretin), neuropeptide Y, cocaine- and amphetamine-regulating transcript, and vasopressin-containing neurons that regulate appetite were spared in posterior nuclei known to participate in feeding regulation.

**Interpretation:**

Degeneration and consequent dysregulation within the hypothalamus relates to significant feeding disturbance in bvFTD. These findings provide a basis for the development of therapeutic models. Ann Neurol 2011

Frontotemporal dementia (FTD) is a progressive neurodegenerative brain disorder. It is the second most common cause of dementia and is as common as Alzheimer disease in individuals with young onset dementia.^1^ Three main clinical phenotypes of FTD are generally recognized based on clinical symptomatology at presentation: behavioral-variant FTD (bvFTD), semantic dementia, and progressive nonfluent aphasia.^2^ Each presentation is characterized by a specific pattern of brain atrophy that concentrates in the frontal and anterior temporal lobes, although overlap occurs across presentation with disease progression. Neuropathologically, most cases of frontotemporal lobar degeneration (FTLD) show intracytoplasmic protein deposition of either the microtubule-associated phosphoprotein tau (FTLD-tau), or the TAR-DNA–binding protein 43 (FTLD-TDP).^3^–^6^ Clinical presentation, however, remains an imperfect predictor of underlying neuropathology.

The behavioral variant is the most common phenotype of FTD, accounting for >50% of all FTD cases. It is characterized by changes in personal, social, and interpersonal conduct, early emotional blunting, and loss of insight.^2^ Abnormal eating behaviors (eg, increased food intake, changes in food preferences)^7^ are present in >60% of cases at presentation but affect >80% over the course of the disease.^8^ Weight gain is common, although patients tend not to become morbidly obese, suggesting changes in metabolism regulation. The biological causes of feeding disturbance in FTD remain poorly understood.

The hypothalamus plays a critical role in feeding regulation.^9^ This structure comprises a number of distinct but interconnected nuclei that directly receive and integrate metabolic input from the periphery, as well as indirect reward, motivation, and sensory input from the cortex,^10^ and then send signals to many regions of the central nervous system regulating food intake and body weight.^9^,^11^ Two appetite-modulating pathways are present: an appetite stimulating pathway, which starts with an empty stomach secreting the hormone ghrelin to target neurons of the arcuate nucleus of the hypothalamus that contain neuropeptide Y (NPY) and agouti-related protein (AGRP), and an appetite-suppressing pathway that starts with adipocytes secreting the hormone leptin to target arcuate neurons containing pro-opiomelanocortin and the cocaine- and amphetamine-regulated transcript (CART; Fig 1). Activation of these pathways orchestrates a series of responses mediated by downstream centers including the paraventricular nucleus (PVN) to control thyroid hormone secretion, feeding behavior, and energy conservation, and by lateral hypothalamic (LH) orexin-producing neurons to control arousal responses and feeding behavior (see Fig 1).^12^ The hypothalamus in FTD has received only limited attention^13^; its contribution to eating disturbance in FTD has not been studied to date, and may have relevance for disease management. This study aimed to determine changes in the hypothalamus in FTD by combining neuroimaging and postmortem investigations.

**FIGURE 1:**
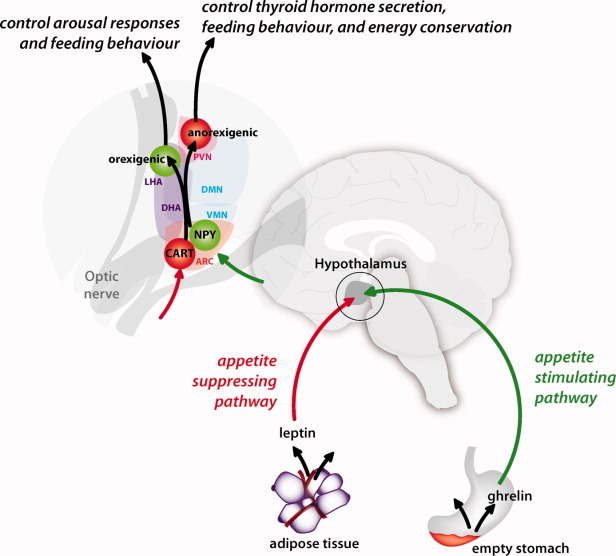
Diagrammatic representation of the appetite-stimulating (green) and appetite-suppressing (red) pathways between the periphery and the hypothalamus, and of their connections within hypothalamic nuclei. PVN = paraventricular nucleus; LHA = lateral hypothalamic area; DMN = dorsomedial nucleus; DHA = dorsal hypothalamic area; VMN = ventromedial nucleus; NPY = neuropeptide Y; CART = cocaine- and amphetamine-regulated transcript; ARC = arcuate nucleus.

## Subjects and Methods

### Cohorts

Two separate cohorts of FTD patients were studied (recruitment details and selection criteria are described in the Supplementary Information) using methods approved by the University of New South Wales Human Ethics Committee. The neuroimaging cohort comprised 18 patients who met clinical diagnostic criteria for bvFTD^2^ based on neurological and cognitive examination at baseline, within 24 months of diagnosis. Sixteen education-matched healthy controls were also enrolled. The postmortem FTD cohort was selected at death for parallel clinicopathological studies. It comprised 12 bvFTD, 6 with FTLD-tau Pick body inclusions and 6 with FTLD-TDP motoneuronlike inclusions (type 2)^14^ without additional neuropathologies, and 6 healthy controls free of significant neuropathology (Table 1). In the neuroimaging cohort, patients with bvFTD were significantly younger than the controls and showed lower performance on cognitive tasks (Mini Mental State Examination [MMSE] and Addenbrooke Cognitive Examination-Revised [ACE-R]) compared to controls. Sex distribution did not differ across groups, although females outnumbered males in the control group, and the reverse pattern was found in the bvFTD. In the postmortem cohort, the bvFTD and control groups were well matched for age, sex, and postmortem delay (see Table 1).

**TABLE 1:** Clinical Characteristics of the Neuroimaging and Postmortem Cohorts (mean ± standard deviation)

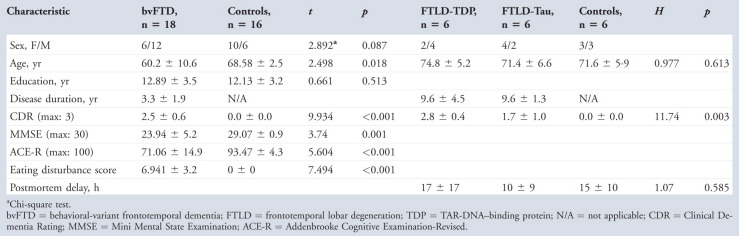

### Characterization of Eating Behavior

Changes in eating behavior were established using the Cambridge Behavioral Inventory (CBI)^15^ in the neuroimaging cohort. This carer questionnaire determines the presence and severity (frequency) of a number of behavioral features using a 5-point scale ranging from 0 (never) to 4 (constantly). A composite eating disturbance score was derived by summing the scores from the relevant CBI items.

### Neuroimaging and Region of Interest Analysis

All participants underwent a structural magnetic resonance imaging (MRI) of the brain. A high-resolution (voxel size: 1 × 1 × 1 mm) T1 image sequence was acquired to conduct morphometric analyses of the hypothalamus. A second sequence (dual T2 images) was collected to measure intracranial volume to correct for interindividual head size differences (see Supplementary Information for imaging sequence details). The hypothalamus was traced manually on the T1 images in the coronal plane using well-defined boundaries.^16^ Given its structural complexity and functional specificity, the obtained hypothalamic volume was divided into 2 equal volumes in the anterior-posterior axis (see Supplementary Information for detailed description of the tracing protocol and Supplementary Fig 1). Initial training was carried out on an independent set of 5 cases. High level of tracing reproducibility of the hypothalamus volume was achieved (intraclass correlation = 0.964). Anterior and posterior hypothalamic volumes were expressed as a proportion of intracranial volume to correct for individual and sex differences in brain size.

### Postmortem Tissue Preparation

Our previous studies have revealed no asymmetry in the cellular components of the hypothalamus.^17^,^18^ As such, coronal tissue blocks of either the left or right hypothalamus were dissected for each case for measurements. Absence of left/right hypothalamic asymmetry was confirmed in this sample for all volumetric measurements and cellular counts (all *p* values > 0.30). Each tissue block was cryoprotected in sucrose solution for 3 to 5 days before serially sectioning at 50μm on a cryostat. Every 15th section (750μm apart) was mounted onto gelatinized slides and stained with 0.5% aqueous cresyl violet and 0.1% Luxol fast blue for cellular quantitation and fiber tract recognition. Six other randomly selected series of sections were immunohistochemically stained with antibodies against tau, TDP-43, NPY, orexin, CART, and vasopressin (Supplementary Table).

### Regional and Cellular Analysis

On each slide, the hypothalamus was traced manually using similar regional boundaries defined in the neuroimaging protocol. Anterior and posterior regions were subdivided where the paraventricular nucleus and the fornix were aligned horizontally. The optical fractionator method was used to count neurons with nucleoli and glial cells in the 50μm-thick Nissl-stained serial sections.^19^ All measurements were performed on blind-coded slides.

### Statistical Analyses

In the neuroimaging cohort, group differences between bvFTD and healthy controls in hypothalamic volumes were investigated using parametric independent sample *t* tests. Within the bvFTD group, the relations between hypothalamic volumes and eating disturbance were examined by contrasting hypothalamic volumes in high and low scorers on the eating behavior composite score, as the distribution of scores was bimodal. Information on feeding was not available for 1 bvFTD patient. In the postmortem cohort, given the small sample size, group differences were tested using nonparametric statistics: either Mann-Whitney tests for 2 groups, or Kruskal-Wallis test for 3 groups followed by post hoc Mann-Whitney tests to determine differences between subgroups.

## Results

### Eating Disturbances in FTD

Features of eating disturbance, such as increased appetite, preference for sweet foods, and increased tendency to eat the same foods, were present only in the bvFTD group and were not observed in the healthy controls (see Table 1). Within the bvFTD group, 10 patients, who displayed the most frequent and severe features, were grouped into a high eating disturbance group (defined by median split). The 2 bvFTD subgroups did not differ with respect to sex distribution, age, disease duration, or MMSE or ACE-R scores.

### Relations to Hypothalamic Changes In Vivo

**FIGURE 2:**
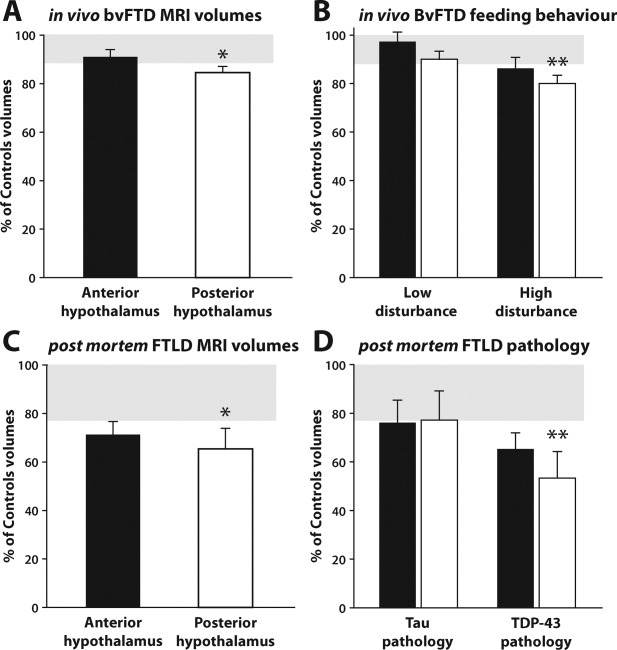
Volumetric changes in the hypothalamus of patients with behavioral-variant frontotemporal dementia (bvFTD) compared to healthy controls. Volumes are standardized to a percentage of the mean hypothalamic volume of the healthy control group (the *gray band* reflecting the standard deviation). (A) In vivo anterior and posterior hypothalamic volumes in bvFTD corrected for head size. Significant atrophy in the posterior hypothalamus atrophy is observed in bvFTD at presentation. (B) In vivo hypothalamic volumes in bvFTD patients exhibiting high or low feeding disturbance corrected for head size. Greater atrophy of the posterior hypothalamus (*white bar*, right) is present in bvFTD patients with high feeding disturbances at presentation compared to bvFTD patients with low feeding disturbance and compared with healthy controls. (C) Postmortem absolute anterior and posterior hypothalamic volumes in frontotemporal lobar degeneration (FTLD). Significant posterior hypothalamic atrophy in FTLD is observed. (D) Postmortem absolute hypothalamic volumes in FTLD patients exhibiting different inclusion pathologies. More severe posterior hypothalamic atrophy is observed in the FTLD-TDP group (*white bar*, right) compared with the FTLD-tau group, and compared with healthy controls. MRI = magnetic resonance imaging; TDP-43 = TAR-DNA–binding protein 43. *Posterior hypothalamus in this group is significantly smaller than that of healthy controls; ** posterior hypothalamus in this group is significantly smaller than those of the other two groups.

Overall, bvFTD patients had smaller hypothalamic volumes than controls (Fig 2), with atrophy most pronounced posteriorly (reduced by 15%, *p* < 0.001) and not significant anteriorly (*p* = 0.08). Within the bvFTD group, patients with a high eating disturbance score exhibited greater posterior hypothalamic atrophy compared with the other bvFTD patients (30% vs 10% reduction in volume, *t*[16] = 2.17, *p* = 0.045; see Fig 2). Although the difference in sex distribution between patient and control groups approached significance, no statistical differences were found in the effect of sex on hypothalamic volumes (all *p* values >0.10).

### Pathological Changes in the Hypothalamus in FTLD

**FIGURE 3:**
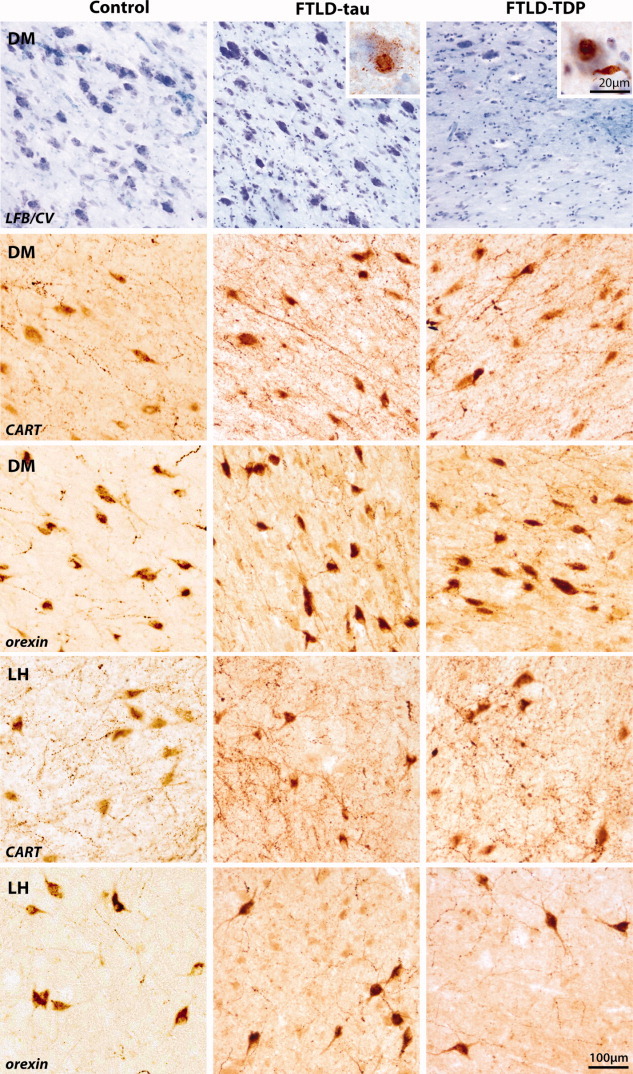
Comparisons of the posterior hypothalamic regions (the dorsomedial [DM] and lateral hypothalamus [LH] nuclei) involved in appetite stimulation between controls and frontotemporal lobar degeneration (FTLD) cases with tau or TAR-DNA–binding protein (TDP) pathology reveals neuronal loss in FTLD-TDP compared to both controls and FTLD-tau *(row 1)* in Nissl-stained sections (0.5% aqueous cresyl violet [CV] and 0.1% Luxol fast blue [LFB]). Insets illustrate tau *(column 2)* and TDP-43 *(column 3)* immunopositive inclusion pathology observed in these regions. Immunoperoxidase staining with cocaine- and amphetamine-regulating transcript (CART, *rows 2 and 4*) and orexin *(rows 3 and 5)* reveal no evident differences in the density or morphology of neurons containing these neuropeptides across groups in either the DM (rows 2 and 3) or the LH *(rows 4 and 5)*.

Patients with FTLD-tau had more abnormal protein deposition in the hypothalamus compared with patients with FTLD-TDP. In FTLD-TDP, sparse TDP-immunoreactive neurites were observed, with occasional intracytoplasmic inclusions in posterior hypothalamic neurons (inset in Fig 3). In patients with FTLD-tau, tau-immunoreactive neurons and neurites were observed throughout the hypothalamus, with occasional Pick body inclusions (see inset in Fig 3).

### Comparison to In Vivo Changes and Assessment of Centers Involved in Feeding Regulation

**FIGURE 4:**
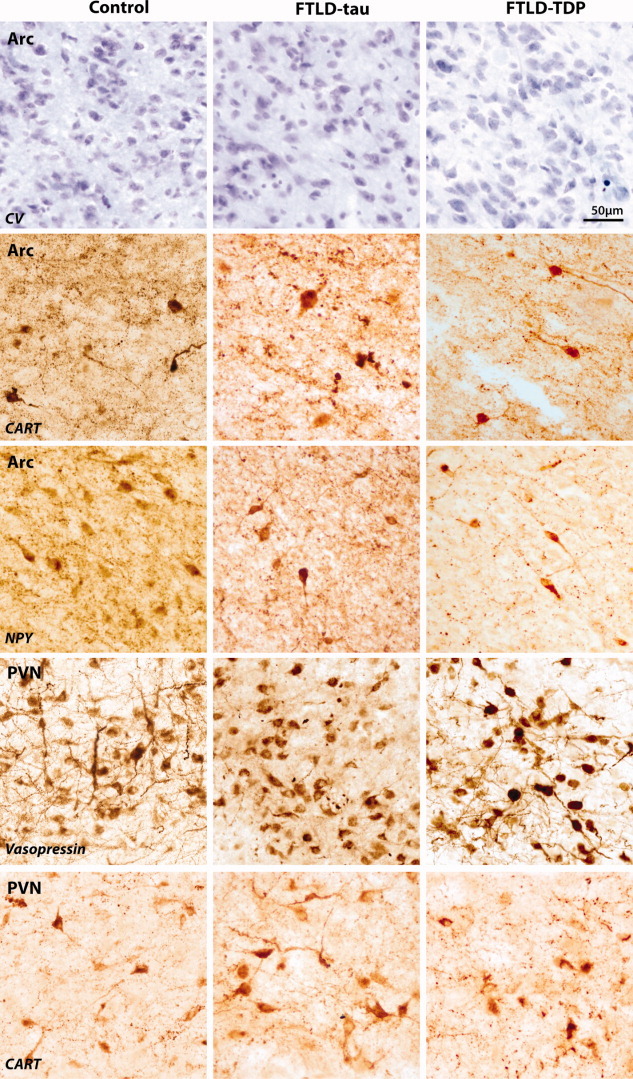
Comparison of the arcuate nucleus (Arc) between controls and frontotemporal lobar degeneration (FTLD) cases with tau or TAR-DNA–binding protein (TDP) pathology. Nissl staining (0.5% aqueous cresyl violet [CV] and 0.1% Luxol fast blue) reveals no noticeable differences in neuron density in Arc *(row 1)*. Immunoperoxidase staining for cocaine- and amphetamine-regulating transcript (CART, *row 2*) within appetite-suppressing neurons and neuropeptide Y (NPY, *row 3*) within appetite-stimulating neurons show no noticeable degeneration of these neurons across groups. Similarly, there were no apparent differences in the staining for vasopressin and CART in the periventricular nucleus (PVN) between the groups.

As found in vivo, postmortem bvFTD cases had significant atrophy of the posterior (volume reduced by 35%; *U* = 8.0, *z* = −2.08, *p* = 0.040; see Fig 2) but not the anterior (*U* = 9.0, *z* = −1.96, *p* = 0.055) hypothalamus compared to controls. Group analyses based on pathology revealed that, despite a similar disease duration, the volume loss arose from more severe atrophy in the FTLD-TDP than in the FTLD-tau group compared to controls (46% vs 22% reduction in volume; Table 2). The severe posterior hypothalamic atrophy in the FTLD-TDP group was associated with neuronal loss and sparse pathology (see above). The low pathology burden in the FTLD-TDP reflects the severity of the neuronal loss in this group. No significant neuronal loss was present in the FTLD-tau group compared with controls (*U* = 4.0, *p* = 0.095). In contrast, the number of anterior hypothalamic neurons and the total estimated number of glial cells in the hypothalamus did not differ across groups. Assessment of the centers involved in the regulation of feeding (see Fig 1) revealed no obvious change in the density or morphology of NPY- or CART-immunoreactive neurons in arcuate nucleus in the anterior hypothalamus (Fig 4). Surprisingly, substantive changes in the density or morphology of orexin- and CART-immunoreactive neurons in LH or other regions of the posterior hypothalamus, such as the dorsomedial nucleus, were not found despite significant loss of posterior hypothalamic neurons (see Fig 3). Similarly, there were no noticeable changes in the density or morphology of vasopressin-immunoreactive neurons in the PVN (see Fig 4).

**TABLE 2:** Hypothalamic Volumes and Neuronal and Glial Counts in FTLD-TDP, FTLD-Tau, and Healthy Controls (mean ± standard deviation)

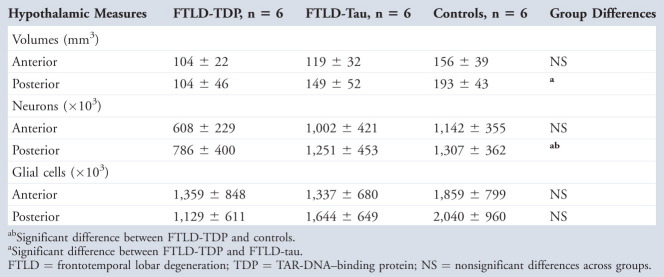

## Discussion

This study reveals a number of novel findings regarding changes in hypothalamus integrity and its relations to eating behavior in FTD. These results have important clinical and theoretical implications and provide insight into the explanation of a distressing feature of FTD that has received surprisingly little attention. As hypothesized, we demonstrated that patients with bvFTD exhibit significant atrophy of the hypothalamus. Using structural neuroimaging, we showed that this atrophy is an early feature of the disease, being already present within 2 years of diagnosis. Our analyses further revealed that this atrophy was most pronounced posteriorly, a region containing nuclei that play a critical role in regulating feeding behavior. In addition, atrophy in the posterior hypothalamus was related to eating behavior in the bvFTD group; patients with high feeding disturbance showed a significantly smaller posterior hypothalamus compared to those with less feeding problems. In contrast, the anterior portion of the hypothalamus was not related to eating behavior in this group. This finding has important implications, as it supports the view that atrophy of specific nuclei in the hypothalamus contributes to the clinical manifestation of feeding disturbance in bvFTD.

Previous structural neuroimaging studies have highlighted that disturbance in an orbitofrontal-insular-striatal brain network underlies the emergence of eating disturbance in FTD.^20^–^22^ Surprisingly, the hypothalamus, which plays a critical role in feeding regulation and regulates other functions central for our metabolic needs (body temperature, water balance, sleep cycle), has not been implicated. Failure to detect changes in the hypothalamus in previous studies was almost certainly methodological. Here, we used region-of-interest identification with intracranial correction, unlike previous works, which have used voxel-based morphometry (VBM), an automated method that investigates differences between groups at the level of individual voxels. Although a powerful approach to examine whole-brain effects without a priori hypotheses, VBM is susceptible to artifacts, particularly in populations with significant cortical atrophy, as is the case with FTD, and has reduced power in detecting changes in small brain structures.^23^

Importantly, the presence of hypothalamic atrophy in FTD was confirmed in a second cohort using a different methodology. Postmortem tissue investigations showed that the pattern of atrophy, with a more pronounced change in the posterior than the anterior hypothalamic region, was identical to that reported on MRI. The severity of atrophy between the 2 studies differed, however; atrophy in the postmortem cohort was at least twice the magnitude of that found in the MRI cohort. In the neuroimaging cohort, hypothalamic measurements were obtained within 2 years of diagnosis, whereas measurements on postmortem tissue were conducted after a disease duration averaging 9 to 10 years. This finding suggests that, although an early feature of the disease, atrophy of the hypothalamus is a continuing process during the course of the disease rather than being limited to the initial phase of the illness. Interestingly, the site of hypothalamic atrophy remains relatively consistent and focused posteriorly over the disease course.

The inclusion pathologies underlying the clinical presentation of bvFTD are variable and remain difficult to predict in life, as clinical cases are evenly distributed between tau- and TDP-positive inclusions.^24^,^25^ Our postmortem analyses, however, demonstrated that hypothalamic atrophy was much more severe in cases with TDP-positive inclusions compared to cases with tau-positive inclusions. This atrophy was also accompanied by significant neuronal loss in the TDP group only, and concentrated in nuclei known to regulate feeding behavior.^26^,^27^ Additional evidence of selective changes in hypothalamic feeding behavior in FTLD-TDP and not FTLD-tau comes from a recent biomarker study showing increases in cerebrospinal fluid AGRP only in FTLD-TDP cases.^28^ Although information on eating behavior was not available in either postmortem cohorts, these results, together with the neuroimaging findings, suggest that eating disturbance in FTD is likely to reflect the presence of TDP rather than tau pathology. Further studies are necessary to confirm these findings.

Despite the considerable posterior cell loss in the hypothalamus in the FTLD-TDP group, no apparent loss of orexin- or CART-containing neurons was present in nuclei that regulate feeding behavior. The loss of the nonpeptidergic neurons in these posterior hypothalamic nuclei may suggest a loss of internal inhibitory regulation leading to overactivity of these peptidergic pathways and the consequent feeding disturbances in bvFTD. Similar disruption of the dorsomedial hypothalamic nucleus in association with hyperactivity of the orexin neurons in LH has been proposed to underlie the binge feeding in attention-deficit/hyperactivity disorder.^29^ The combination of pathology-specific neuronal loss together with the preservation of neurons containing peptides involved in feeding regulation may have considerable clinical implications as targeted therapeutic interventions are developed.

One of the strengths of this study is the combination of clinical information and in vivo imaging techniques to measure changes in the hypothalamus plus postmortem confirmation of the findings in an independent sample. The addition of a postmortem tissue arm to the study allowed the investigation of specific neuron populations and peptides, which are too small to be detected and measured with neuroimaging. The use of different sample populations of clinically diagnosed patients and neuropathologically confirmed cases is not ideal and raises interesting questions regarding the underlying pathology in the cases enrolled in the neuroimaging study. The pathology study suggests that patients with TDP-43 inclusions may be particularly prone to eating disturbance. Longitudinal investigations will be necessary to obtain clinicopathological information on the same cases. All neuroimaging cases were assessed by the same neurologist (J.R.H.), who has extensive experience in the early clinical diagnosis of FTD. In addition, we were careful in the FTD case selection to include only patients who met current diagnostic criteria unequivocally, showed clear cognitive deficits on neuropsychological examination, and exhibited cortical changes on MRI consistent with the disease. The presence of MRI abnormality is particularly important, reducing the likelihood of including so-called phenocopy cases.^30^ Longitudinal analyses will also clarify the pattern of eating disturbance with disease progression. Our postmortem analyses indicate that atrophy of the hypothalamus is not confined to the early phase of the illness, but is progressive over the course of the disease. Clinically, however, little information is available about changes in eating behavior over time once the initial disturbance has been documented. Importantly, disease progression in bvFTD is accompanied by increasing apathy.^31^ It is therefore plausible that abnormal eating behavior may become less salient as apathy becomes more prominent.

In conclusion, this study is the first to link atrophy and pathology in the hypothalamus with eating disturbance in bvFTD. Behaviorally, patients with a high level of eating disturbance exhibited significant atrophy of the posterior hypothalamus. Combined with the postmortem investigations, our findings indicate that this eating disturbance is related to neuronal loss in this region and is greater in patients with TDP-43 pathology. A recent study^28^ supports the view that hypothalamic changes appear specific to FTLD-TDP. Further studies are necessary to clarify the contributions of other brain regions to eating disturbance in FTLD. Overall, the present study shows that degeneration and consequent dysregulation within the hypothalamus relates to significant feeding disturbance in bvFTD, providing a basis for the development of therapeutic models for this clinical feature.

## Potential Conflicts of Interest

O.P. and J.R.H. have grants pending from the National Health and Medical Research Council of Australia.
