# Supplementary figures and images for: Eating and hypothalamus changes in behavioral-variant frontotemporal dementia
Source: Ann Neurol. 2011 Feb;69(2):312–9. doi: 10.1002/ana.22244 (PMC3084499; doi:10.1002/ana.22244)

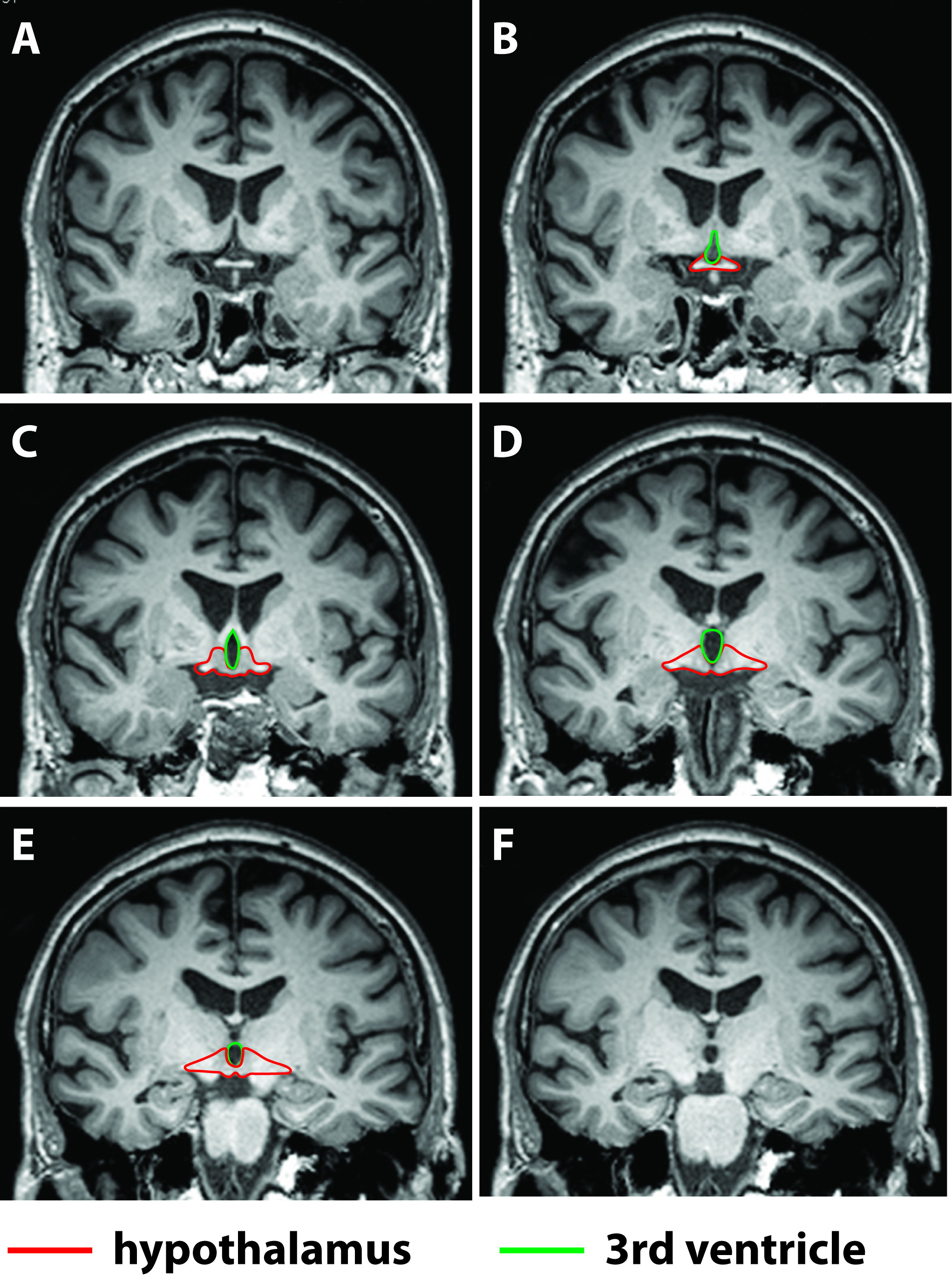

Supplement: Supplementary file 1 [file ana0069-0312-SD1.tif]
