# Supplementary material for: Eating and hypothalamus changes in behavioral-variant frontotemporal dementia
Source: Ann Neurol. 2011 Feb;69(2):312–9. doi: 10.1002/ana.22244 (PMC3084499; doi:10.1002/ana.22244)
Supplement: Supplementary file 2 [file ana0069-0312-SD2.doc]

**Supplementary Information**

**Patient recruitment and general procedure**

For the neuroimaging cohort, the 18 bvFTD patients were selected at their earliest presentation for clinical classification and evaluation of eating disturbances from consecutive bvFTD patients referred for diagnosis to the Frontier Research Program between January 2008 - June 2009 (N=31). All patients were examined by the same experienced neurologist (JRH) and met clinical diagnostic criteria for bvFTD^1^ based on neurological and cognitive examination at baseline, evidence of change on structural MRI and with onset of disease within 24 months of presentation. Patients were selected only if: **(i)** a diagnosis of bvFTD was unequivocal (i.e., no secondary diagnosis), **(ii)** they met none of the exclusion criteria below; and **(iii)** their MRI scan was of good enough quality to allow tracings of hypothalamus (i.e., with minimal movement artefact). The healthy controls were recruited from Neuroscience Research Australia brain donor program (N=150) and from local community clubs, matching to the cases for age, sex and education level as much as possible. Controls all scored between 27 and 30 on the Mini Mental State Examination (MMSE), 0 on the Clinical Dementia Rating (CDR)^2^, and above 85 on the Addenbrooke Cognitive Examination-Revised (ACE-R).^3^ Medical history, current medication use and presence of depression symptomatology^4^ were also assessed. Exclusion criteria included prior history of mental illness, significant head injury, movement disorders, cerebrovascular disease, alcohol and other drug abuse, use of psychotropic medication, and limited English proficiency. Because of the MRI procedure, individuals with ferromagnetic implants or a prior history of claustrophobia were excluded. Presence of eating disturbance was established by summing the scores from the relevant items from the Cambridge Behavioural Inventory (“She/He prefers sweet foods more than before”; “She/He wants to eat the same foods repeatedly”; “Her/His appetite is greater, she/he eats more than before”)^5^ into a composite eating disturbance score.

For the postmortem cohort, the 12 bvFTD cases (6 with FTLD-Tau and Pick body inclusions and 6 with FTLD-TDP-43 and motorneuron-like inclusions (Type 2) ^6, 7^ and 6 healthy controls were selected from the Sydney Brain Bank under methods approved by the University of New South Wales Human Ethics Committee. Causes of death included cardiopulmonary arrest, pneumonia, heart disease/cardiac arrest, renal failure, pulmonary infarcts, and metastatic ovarian or prostate cancer. Brains were collected at postmortem, formalin-fixed and prepared for volumetric and diagnostic studies, and the remaining tissue stored for research purposes. All patients met clinical diagnostic criteria for bvFTD^1^ based on neurological and cognitive examination, and all met pathological criteria for FTLD^6, 8^and showed no pathological comorbidities. Healthy controls had no neurological or psychiatric disease and were free of any significant neuropathology.

**Neuroimaging protocol**

Two imaging sequences were acquired on a 3-Tesla Achieva Philips scanner using a standard 8-channel head coil. Participants were in the supine position and their head was padded to minimise movements. The first image series was a high-resolution 3D TFE T1 sequence with an inversion pre-pulse at a shot interval of 1800 ms (TR/TE/FL: 6.4/2.8/8 ms; flip angle: 8°; 1 mm isotropic voxel size, 200 coronal slices; field of view 256, 256 x 256 matrix), scan time: 7 min 42 s. This sequence was used to conduct morphometric analyses. The hypothalamus was traced manually on a computer screen at 400% magnification by a single tracer (BYKL) who was blind to group membership using ANALYZE 6.0 (Brain Imaging Resource, Mayo foundation, Rochester, MN) with a pen and digitized tablet. Tracing of the hypothalamus was carried out using well-defined boundaries and excluded the 3^rd^ ventricle.^9^ Given its structural complexity and functional specificity, the hypothalamus was divided into two equal volumes in the anterior - posterior axis.

The second image sequence was a dual TE 18/80 T2 sequence (TR/TE: 2100/18, 80 ms; flip angle 90°; voxel size: 1 x 1 x 3.5 mm, 2 x 40 horizontal slices; field of view 512 x 512), scan time: 4 min 30 s. This sequence was used to measure intracranial volume to calculate corrected hypothalamic volumes. Intracranial volume was obtained by tracing manually the inner surface of the skull on consecutive horizontal images of the T2 sequence. This volume, which included the brain, cerebellum, brain stem and surrounding cerebral spinal fluid, was used to adjust hypothalamic volumes for individual and sex differences in brain size.

**Definitions of hypothalamus boundaries**

The boundaries of the hypothalamus were defined in the coronal plane on the T1 MR images as follows:

**Rostral-caudal axis:** The anterior boundary of the hypothalamus was defined by the anterior margin of optic chiasm, and more specifically, by the first slice on which the optic tract is attached to the brain by two “wisps” of white matter. The posterior boundary was defined by the posterior margin of the mamillary bodies; more specifically, it was defined by the last slice on which the mamillary bodies are clearly visible.

**Dorsal-ventral axis:** The inferior boundary was defined by the medial inferior part of the floor of the diencephalon. In the most anterior slices (generally the first 3-4 images), the superior border was defined by the anterior commissure. In the remaining, posterior slices, the boundary was defined by the hypothalamic sulcus.

**Medial-lateral axis:** The lateral border extended from the most lateral point of the optic tract to the base of the internal capsule (anteriorly) or to the hypothalamic sulcus (posteriorly).

For the MR volumes, the hypothalamus and third ventricle were delineated together. Following tracing completion, the volume of the 3^rd^ ventricle was subtracted from the total volume traced. The optic tract was included in the final volume in order to improve tracing consistency and reliability of the MR volumes.

*** INSERT SUPPLEMENTARY INFORMATION FIGURE 1 ABOUT HERE ***

**Cellular Nissl staining**

For Luxol fast blue/cresyl violet (LFB/CV) staining, one series of 50 µm thick coronal sections cut 750 µm apart throughout the hypothalamus were first mounted on glass slides. They then underwent delipidation by passing through a series of alcohol and xylene baths before being stained with 0.1% LFB (Solvent Blue 38, Sigma) over night at room temperature. On the following day, the slides were rinsed with H_2_0 till no excess staining remained, before the LFB was stabilized with 500mg/1000ml lithium carbonate (Sigma-Aldrich). The slides were rinsed with H_2_O to remove the excess lithium carbonate and stained with 0.5% cresyl violet (Sigma) in 10% acetic acid solution for 1-2 minutes. After dehydrating the LFB/CV stained sections were cover-slipped with DPX mounting media (Sigma).

**Immunohistochemistry**

Six series of free floating 50 µm thick sections (750 µm apart) were immunohistochemically processed with primary antibodies against tau, TDP-43, NPY, orexin, CART and vasopressin (see supplementary information table 1 for details of antibodies).

**Supplementary Information Table 1. Details of immunohistochemistry for peptide staining in the postmortem cohort**

| **Primary Antibody** | **Company (Product #)** | **Host** | **Primary Antibody Dilution** | **Blocking serum** | **Secondary Antibody** |
| --- | --- | --- | --- | --- | --- |
| **Neuropeptide Y (NPY)** | J. Oliver | Sheep | 1:50,000 | Rabbit | Biotinylated rabbit anti-sheep (BA 6000 Vector Laboratories) |
| **Orexin** | Phoenix Pharmaceuticals (H-003-30) | Rabbit | 1:30,000 | Goat | Biotinylated goat anti-rabbit (BA 1000 Vector Laboratories) |
| **Cocaine- and amphetamine regulated transcript (CART)** | M. Kuhar | Rabbit | 1:10,000 | Goat | Biotinylated goat anti-rabbit (BA 1000 Vector Laboratories) |
| **Vasopressin** | Millipore | Rabbit | 1: 30,000 | Goat | Biotinylated goat anti-rabbit (BA 1000 Vector Laboratories) |

The brain sections were first thoroughly rinsed three times with 50% ethanol solution for 15 minutes, and any non-specific peroxidase quenched in 3% H_2_O_2_ and 50% ethanol for 20 minutes. After the quenching step the sections were rinsed three times with potassium phosphate buffered saline (KPBS). To avoid nonspecific binding, the sections were incubated in KPBS containing 5% serum (from the same species as the corresponding secondary antibody was raised in) for 20 minutes. The sections were then incubated overnight at room temperature in primary antibody solution (5% serum in KPBS). The following day, the sections were rinsed two times in KPBS and once with 2% serum in KBS for 15 minutes before incubation with biotinylated secondary antibody for one hour at room temperature in 1:200 dilation. After rinsing the section three times with KPBS for 15 minutes, the sections were incubated with avidin-biotin-peroxidase complex (ABC elite kit, Vector Laboratories) prepared in KPBS for one hour at room temperature. Once the incubation was completed the sections were rinsed three times with KPBS, and the peroxidase reaction developed by incubation in 25mg/ml 3,3’-diaminobenzidine for 10 minutes and 0,003% H_2_O_2_ for 5 minutes. The sections were mounted onto chrome-alum gelatinized slides. Once the sections were dried, slides were passed through a series of alcohol and xylene baths for delipidation and cover-slipped with DPX mounting media (Sigma). Negative controls were performed in which tris buffer replaced the primary antibody in the staining process. No staining was observed in these sections.

**Regional and cellular analysis**

Imaging and quantification were performed on blind coded hypothalamic tissue sections using an Olympus microscope fitted with a CCD camera connected to a PC running Stereo Investigator 8.0 (MBF Bioscience, Microbrightfield Inc., USA). The same borders of the hypothalamus used in the MRI delineations were defined using a 4X objective and traced. The white matter bundles within the hypothalamus were also traced and excluded and the hypothalamus volume calculated using Cavalieri’s principle (cross-sectional area measured x distance between serial sections). Training was performed using a separate set of hypothalamic sections from control cases (3 brains traced 5 times each) and resulted in an intra-class correlation for the hypothalamus volume of 0.995. Anterior and posterior regions were subdivided when the paraventricular nucleus and the fornix were aligned horizontally.

Estimations of the total number of neurons and glial cells were performed in the 50 μm thick Nissl stained serial sections of the hypothalamus using an unbiased stereological quantification method with the optical fractionator principle.^10^ Based on their morphological appearance, neurons were defined by a transparent cytoplasm and relative large nuclei with a nucleolus, and glial cells were defined by dark stained cytoplasm and small nuclei. Small heavy stained cells were considered oligodendrocytes and were not included in the counting. The optical frame size was 1530 μm^2^ with an x/y length of 1010 μm and a disector volume of 76500 μm^3^. Cell counting was performed using Sterero Investigator 8.0 with the average variability in repeated measures (2 brains counted 5 times each) of 9.3% for neuronal density, 2.4% for glial density and 4.2% for estimated cell numbers (< 7% in each individual case). For the study, the number of disectors assessed on average was 316/case (range 211-443). The average number of neurons counted per case was 180 (range 115-305) and the average coefficient of error for sampling density per case was 0.024 (SD 0.010). The average number of glia counted/case was 263 (range 69-420) and the average coefficient of error for sampling density per case was 0.026 (SD 0.011).

**Supplementary Information References**

1. Neary D, Snowden JS, Gustafson L et al. Frontotemporal lobar degeneration: a consensus on clinical diagnostic criteria. Neurology. 1998;51:1546-1554

2. Morris JC. The Clinical Dementia Rating (CDR): current version and scoring rules. Neurology. 1993;43:2412-2414

3. Mioshi E, Dawson K, Mitchell J et al. The Addenbrooke's Cognitive Examination Revised (ACE-R): a brief cognitive test battery for dementia screening. Int J Geriatr Psychiatry. 2006;21:1078-1085

4. Lovibond PF, Lovibond SH. The structure of negative emotional states: comparison of the Depression Anxiety Stress Scales (DASS) with the Beck Depression and Anxiety Inventories. Behav Res Ther. 1995;33:335-343

5. Bozeat S, Gregory CA, Ralph MA, Hodges JR. Which neuropsychiatric and behavioural features distinguish frontal and temporal variants of frontotemporal dementia from Alzheimer's disease? J Neurol Neurosurg Psychiatry. 2000;69:178-186

6. Mackenzie IR, Neumann M, Bigio EH et al. Nomenclature and nosology for neuropathologic subtypes of frontotemporal lobar degeneration: an update. Acta Neuropathol. 2010;119:1-4

7. Sampathu DM, Neumann M, Kwong LK et al. Pathological heterogeneity of frontotemporal lobar degeneration with ubiquitin-positive inclusions delineated by ubiquitin immunohistochemistry and novel monoclonal antibodies. Am J Pathol. 2006;169:1343-1352

8. Cairns NJ, Bigio EH, Mackenzie IR et al. Neuropathologic diagnostic and nosologic criteria for frontotemporal lobar degeneration: consensus of the Consortium for Frontotemporal Lobar Degeneration. Acta Neuropathologica (Berlin). 2007;114:5-22

9. Jacobson S, Marcus EM. Hypothalamus, neuroendocrine system, and autonomic nervous system. In: Jacobson S, Marcum EM, eds. Neuroanatomy for the neuroscientist. Boston: Springer, 2008:165-187

10. West MJ, Slomianka L, Gundersen HJ. Unbiased stereological estimation of the total number of neurons in the subdivisions of the rat hippocampus using the optical fractionator. Anat Rec. 1991;231:482-497

**Supplementary Information figure legend**

**Figure 1. A-F** Tracing of hypothalamus (red) and the 3^rd^ ventricle (green) in the coronal plane, from anterior to posterior plane.
